# Confinement and entanglement dynamics on a digital quantum computer

**DOI:** 10.1038/s41598-021-90849-5

**Published:** 2021-06-02

**Authors:** Joseph Vovrosh, Johannes Knolle

**Affiliations:** 1 Blackett Laboratory, Imperial College London, London, SW7 2AZ UK; 2 Department of Physics TQM, Technische Universität München, James-Franck-Straße 1, 85748 Garching, Germany; 3 Munich Center for Quantum Science and Technology (MCQST), 80799 Munich, Germany

**Keywords:** Quantum simulation, Quantum fluids and solids

## Abstract

Confinement describes the phenomenon when the attraction between two particles grows with their distance, most prominently found in quantum chromodynamics (QCD) between quarks. In condensed matter physics, confinement can appear in quantum spin chains, for example, in the one dimensional transverse field Ising model (TFIM) with an additional longitudinal field, famously observed in the quantum material cobalt niobate or in optical lattices. Here, we establish that state-of-the-art quantum computers have reached capabilities to simulate confinement physics in spin chains. We report quantitative confinement signatures of the TFIM on an IBM quantum computer observed via two distinct velocities for information propagation from domain walls and their mesonic bound states. We also find the confinement induced slow down of entanglement spreading by implementing randomized measurement protocols for the second order Rényi entanglement entropy. Our results are a crucial step for probing non-perturbative interacting quantum phenomena on digital quantum computers beyond the capabilities of classical hardware.

## Introduction

Quantum computers are proposed to out-perform their classical counterparts for selected applications^1^. It is Richard Feynman’s prediction from 1982 that a quantum device would have the ability to directly simulate quantum systems which has most potential for solving a number of long-standing fundamental problems in science^2–4^, for example in chemistry^5^ or for lattice gauge theories (LGT) relevant in high energy physics^6–8^.

In recent years there has been a tremendous push in order to realise a digital quantum computer, e.g., based on superconducting circuits. Despite these efforts, current working computers are described as Noisy Intermediate-Scale Quantum (NISQ) devices^9^, which do not have enough qubits or small enough errors to perform error correction. The uses of NISQ devices are still in question but we show here that they have reached capabilities for observing quantum confinement physics.

**Figure 1:**
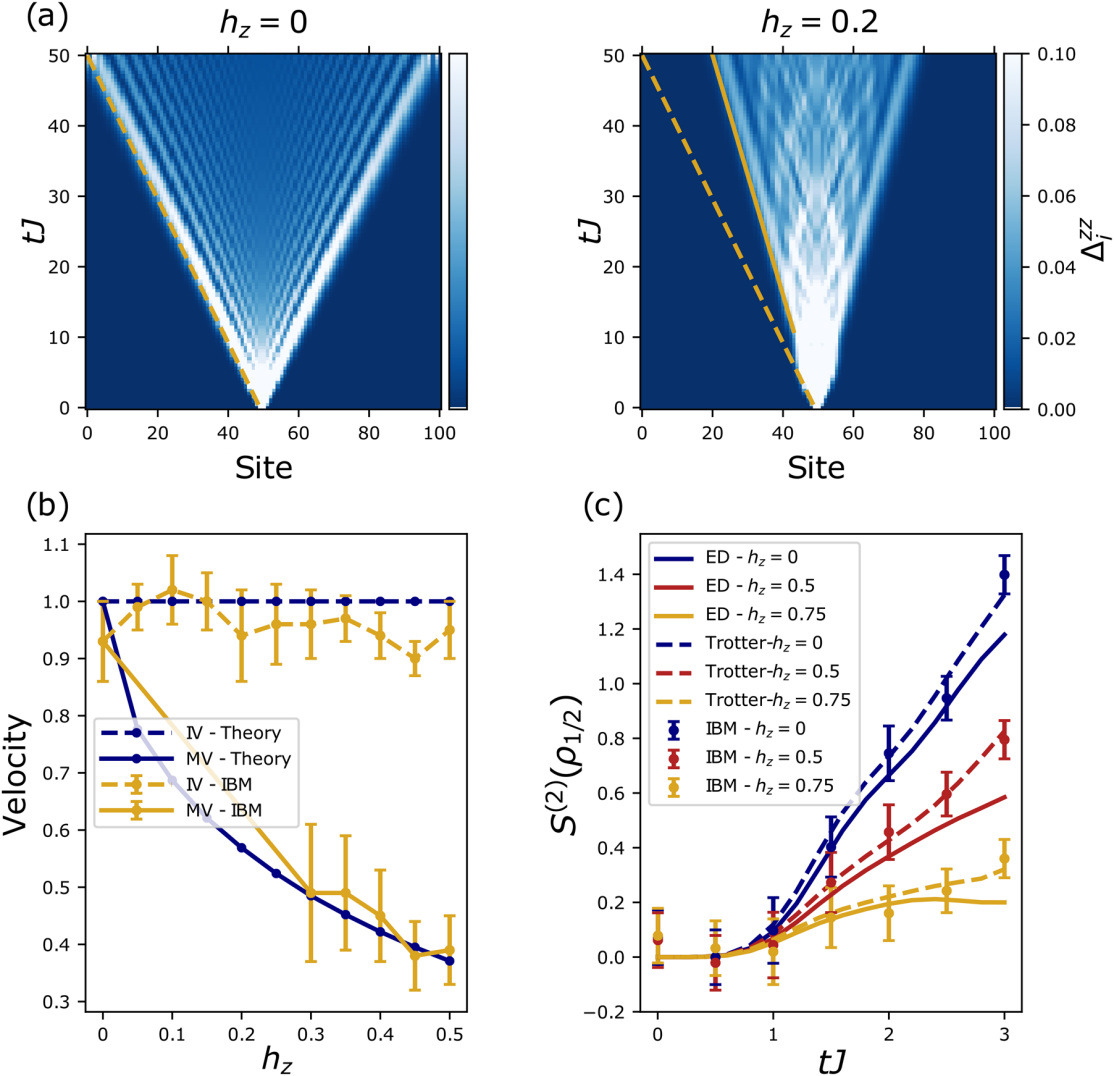
Velocities and entanglement Renyi entropy from the IBM device. **(a)** Real time dynamics of domain wall positions within the two kink subspace following a quantum quench to the TFIM without and with additional longitudinal field $h_z$. These results are calculated via exact diagonalisation of the two kink subspace. Here, $L=101$, $h_x=0.5$ and the initial state is ferromagnetic with a single flipped spin in the centre. For $h_z=0$ the light cone structure of free particles is visible. For the confining case $h_z\ne 0$ two velocities are observable, an initial velocity (dashed) equal to the free case and the meson velocity (solid) at longer times. **(b)** Comparisons of the two velocities, the initial velocity (IV) and meson velocity (MV), as measured on the IBM quantum computer ($h_x=0.5$ and $L=9$) after error mitigation and as theoretically predicted. Error bars displayed are the standard deviation of a range of velocities obtained, more details are given in the Supplementary Material. **(c)** Data from randomized measurements for the half chain second order Rényi entropy after a global quantum quench to the TFIM with varying a longitudinal field strengths on the state $\vert \frac{L}{2}-1,2\rangle$ for $h_x=0.5$, $L=6$. The ballistic entanglement growth of the free case is suppressed because of confinement for increasing longitudinal field. Here, error bars are calculated by jacknife resampling. The inherent error in the IBM device leads to offset which has been removed, see the Supplementary Material for details.

Our basic understanding of confinement in QCD is limited because it is an example of a non-perturbative quantum many body effect. LGT descriptions thereof^10^ are hard to simulate on classical computers and remain beyond the reach of current NISQ devices. As a first step for proving the usefulness of quantum computers as quantum simulators one can study one dimensional lattice systems of condensed matter physics displaying similar confinement physics. Examples include, the transverse field Ising model (TFIM) with long range interactions^11^, the lattice Schwinger model^6^, the XXZ spin-1/2 chain^12^ and, the model considered here, the TFIM with an additional longitudinal field^13–19^. The pure TFIM has free fermion excitations that correspond to domain walls between spin-aligned segments. An additional longitudinal field gives rise to an emergent confining potential between these fermionic excitations which can be described as ‘mesonic’ bound states of domain walls^20,21^. Fig.1(a+b) shows the different velocities of free (dashed) and bound (solid) particles that govern the time evolution of correlation spreading. Together with the confinement induced halting of entanglement spreading, Fig.1(c), this provides direct signatures of confinement physics on a digital quantum computer.

The TFIM has been studied with analytical methods^14,21,22^ but the full time evolution of the non-integrable model with a longitudinal field has been restricted to numerical simulations for limited system sizes or time windows, e.g. with the density matrix renormalization group (DMRG)^18^. In general, out-of-equilibrium quantum dynamics of many-body systems are notoriously difficult to simulate with classical computers because the memory required scales exponentially with system size. In principle, quantum computers are free of such problems, however, available NISQ devices come with their own limitations. Firstly, they only have a restricted number of available qubits. Secondly, their large errors when executing a quantum circuit limit circuit depth and in turn the accessible simulation time. Nevertheless, there have already been promising results for the magnetization dynamics of different spin chains^23–25^. However, up to now the accuracy of the devices was barely enough to qualitatively distinguish genuine interaction from disorder/noise effects^26^. Here, we take the next step and report digital quantum simulation of confinement in out-of-equilibrium dynamics of spin chains of up to nine spins on the latest IBM machines code-named Boeblingen and Paris.

### Model and the two kink subspace

The one dimensional TFIM with an additional longitudinal field is described by the following Hamiltonian

1 $$\begin{aligned} H = -J\bigg [\sum _{i=0}^{L-2}\sigma _i^{z}\sigma _{i+1}^{z} + h_x\sum _{i=0}^{L-1} \sigma _i^{x}+ h_z\sum _{i=0}^{L-1} \sigma _i^{z}\bigg ], \end{aligned}$$

where $\sigma _i^{\alpha }$, $\alpha \in {x\text {, }y\text {, }z}$ are the Pauli matrices acting on the $i$th site, $i \in \{0,1,2,...,L-1\}$, *L* is the length of the chain, *J* is the Ising exchange of nearest neighbour spin 1/2 and $h_{x/z}$ are the relative strengths of the transverse and longitudinal fields. For $h_z = 0$, the TFIM can be exactly diagonalised via Jordan-Wigner transformation and describes free fermions. Here we restrict ourselves to transverse field strengths below its critical value, $h_c = J$, in the ordered phase, where fermions are approximately described by domain wall (or kink) excitations $\vert ...\uparrow \uparrow \downarrow \downarrow ...\rangle$ aligned in the *z* direction. The longitudinal field then gives rise to a confining potential between kinks strongly affecting the non-equilibrium dynamics of the system. An established way to elucidate the confinement physics is to study the dynamics in a restricted two-kink subspace which not only allows us to predict analytically the velocities and masses of the mesons, see Fig. 1, but crucially for this work, it also forms the basis of our error mitigation protocol. We project Eq. (1) into the two kink subspace written in the basis $\vert j,n\rangle = \vert \uparrow \uparrow ...\uparrow \downarrow _j...\downarrow _{j+n-1}\uparrow ..\uparrow \uparrow \rangle$. This gives ${\mathcal {H}} = P^{-1}HP$, in which *P* is the projection operator and (up to constant terms).

2 $$\begin{aligned} {\mathcal {H}} =\sum _{\begin{array}{c} 0\le j<L-1,\\ 0<n<L-j-1 \end{array}} \bigg \{-h_x\big [\vert j,n+1\rangle +\vert j,n-1\rangle +\vert j+1,n-1\rangle +\vert j-1,n+1\rangle \big ]\langle j,n\vert +V(n)\vert j,n\rangle \langle j,n\vert \bigg \}, \end{aligned}$$

with $V(n) = 2h_zn$. The first term of this subspace Hamiltonian is a kinetic term that allows the kinks to ‘hop’, and the second term is the effective potential, *V*(*n*), linearly increasing with kink separation *n*. Thus, the out-of-equilibrium motion of kinks will be similar to that of quarks; pairs of kinks that are produced propagate in opposite directions until the confining potential halts their motion and pulls them back, leading to oscillatory motion, this is what we call a meson. These mesonic bound states of kinks are then able to propagate as a pair with a much slower velocity than if they were free.

### Signatures of confinement

A hallmark signature of confinement is the formation of mesons whose properties, e.g. masses, have been measured with different observables depending on the experimental or numerical feasibility^15–18^. In order to use NISQ devices as new tools for quantum simulations it is necessary to carefully design the measurement set-up to obtain unambiguous signatures for the available system sizes and time windows. Among the many different protocols we have checked, the three following measures can give qualitative as well as quantitative results on the IBM devices.

(i) Confinement physics can be observed in the probability dynamics of kinks as a function of time given by
  3 $$\begin{aligned} \Delta _{i}^{zz}=\langle \psi (t)\vert \frac{1}{2}(1-\sigma ^{z}_{i}\sigma ^{z}_{i+1})\vert \psi (t)\rangle . \end{aligned}$$
  This function gives the local probability of kink position on a chain. Thus, it provides a very clear picture of kink motion. In fact, it not only shows the kinks position with time, it also shows the mesons position once they form. We have benchmarked that both of the two velocities can be extracted from $\Delta ^{zz}_i$ with quantitative agreement to the theory described above.
(ii) Another key signature is the suppression of half chain entanglement entropy spreading due to confinement. In the free case, $h_z=0$, the entanglement entropy is expected to increase linearly^27^. However, with a non-zero confining field $h_z$ this growth is suppressed in a characteristic fashion^18^. In general, entanglement entropy is not easy to calculate on a real quantum computer as it requires some form of state tomography. Here, building on recent progress for randomized measurement algorithms^28^ we are able to measure—for the first time on a digital quantum computer – the second order Réyni entanglement defined as
  4 $$\begin{aligned} S^{(2)}(\rho _A) = - \log _2{\rm{Tr}}(\rho _A^2). \end{aligned}$$
  Here, $\rho _A$ is the reduced density matrix for the half-chain subsystem A. With a repeated measurement of a set of random single qubit gates on each site in the subsystem A, $S^{(2)}(\rho _A)$ can be approximated by
  5 $$\begin{aligned} S^{(2)}(\rho _A) = - \log _2{\bar{X}},\text{ with } X = 2^{N_A}\sum _{s_A,s'_A}(-2)^{D[s_A,s'_A]}P(s_A)P(s'_A), \end{aligned}$$
  where $s_A$ denotes a measurement outcome, $P(s_A)$ is the probability of measuring $s_A$, $D[s_A,s'_A]$ is the Hamming distance between $s_A$ and $s'_A$, $N_A$ is the dimension of *A* and ${\bar{X}}$ denotes the ensemble average of *X* over the set of different random single qubit gates used^28^.
(iii) The last viable diagnostic of confinement are the probability maps of kink positions^12^. After time evolution the probabilities of the first kink position with respect to the position of the second kink is mapped to show that, in the presence of an additional longitudinal field, it is favourable for the two kinks to reside close to each other, i.e. the kinks form a meson.

### Error mitigation and post selection

When implementing dynamics on the IBM quantum computers there are four main sources of error: initialisation and measurement error, single qubit gate error, controlled-NOT (CNOT) gate error and decoherence. A crucial ingredient for obtaining quantitative results are the following error mitigations.

(i) The best subset of qubits is chosen. This is done by calculating the average error for each subset of qubits with the desired topology, a chain of length *L*, within the machine. Error types are not weighted evenly as the gate error is more important than the readout error for the protocols used in this work. Although this method is not scalable with increasing number of qubits, it is well suited for current devices.
(ii) The initial states considered here have an inherent inversion symmetry around the centre site such that the data should reflect this symmetry. However, because of inhomogeneous errors in the quantum computer there are sizeable deviations which we correct by averaging the data and with its mirror image.
(iii) The last and crucial error mitigation technique is the projection of the data into the two kink subspace. For our initial states and quench set-up, the error-free time evolution mainly takes place within this subspace, and crucially, it contains the desired confinement physics. Hence, this post selection to the two kink subspace is a viable tool for eliminating errors, more details are given in the Supplementary Material.

With the error mitigation described above, as well as repeating measurements on different days^26^, simulations for meson velocities and probability maps with up to nine qubits and times of up to $tJ=8$ were obtained. For the second order Rényi entanglement entropy it is necessary to apply random unitaries before measurements such that only mitigation technique i) can be used. Times that can be simulated for the second order Rényi entropy are up to $tJ\sim 3$.

## Results

Data for the probability maps as well as $\Delta ^{zz}_i$ was collected from the IBM computer Boeblingen which has a total of 20 qubits. We employed a global quench protocol from an initially aligned state with a single spin flipped at the centre (the state $\vert \frac{L-1}{2},1\rangle$) to the TFIM with and without a longitudinal field. Randomized measurements for the second order Rényi entanglement entropy were carried out on the IBM device Paris with a total of 27 qubits. We employed a global quench from an initially aligned state with two spins flipped at the centre (the state $\vert \frac{L}{2}-1,2\rangle$). We used open boundary conditions in all cases.

**Figure 2:**
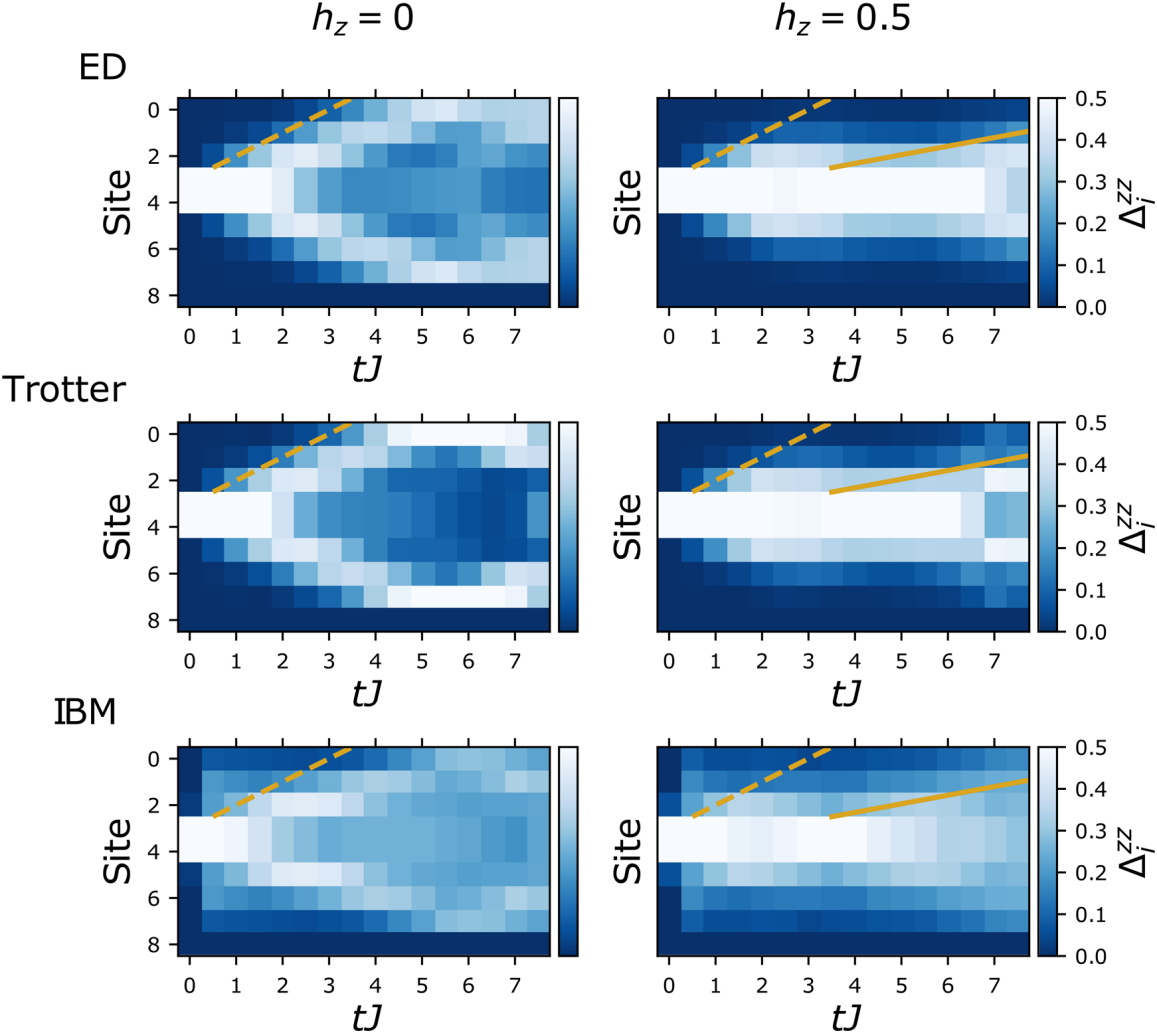
Time evolution of probability dynamics of kinks. Data for $\Delta _{i}^{zz}$ after a global quantum quench to the TFIM with and without a longitudinal field starting from the state $\vert \frac{L-1}{2},1\rangle$. In all presented data $h_x=0.5$ and $L=9$. The graphs on the left show the free kink case, $h_z=0$ and the graphs on the right the confined one $h_z =0.5$. Clear suppression of the kink separation can be seen in the latter as well as the emergence of a second slower velocity – both signatures of confinement.

**Figure 3:**
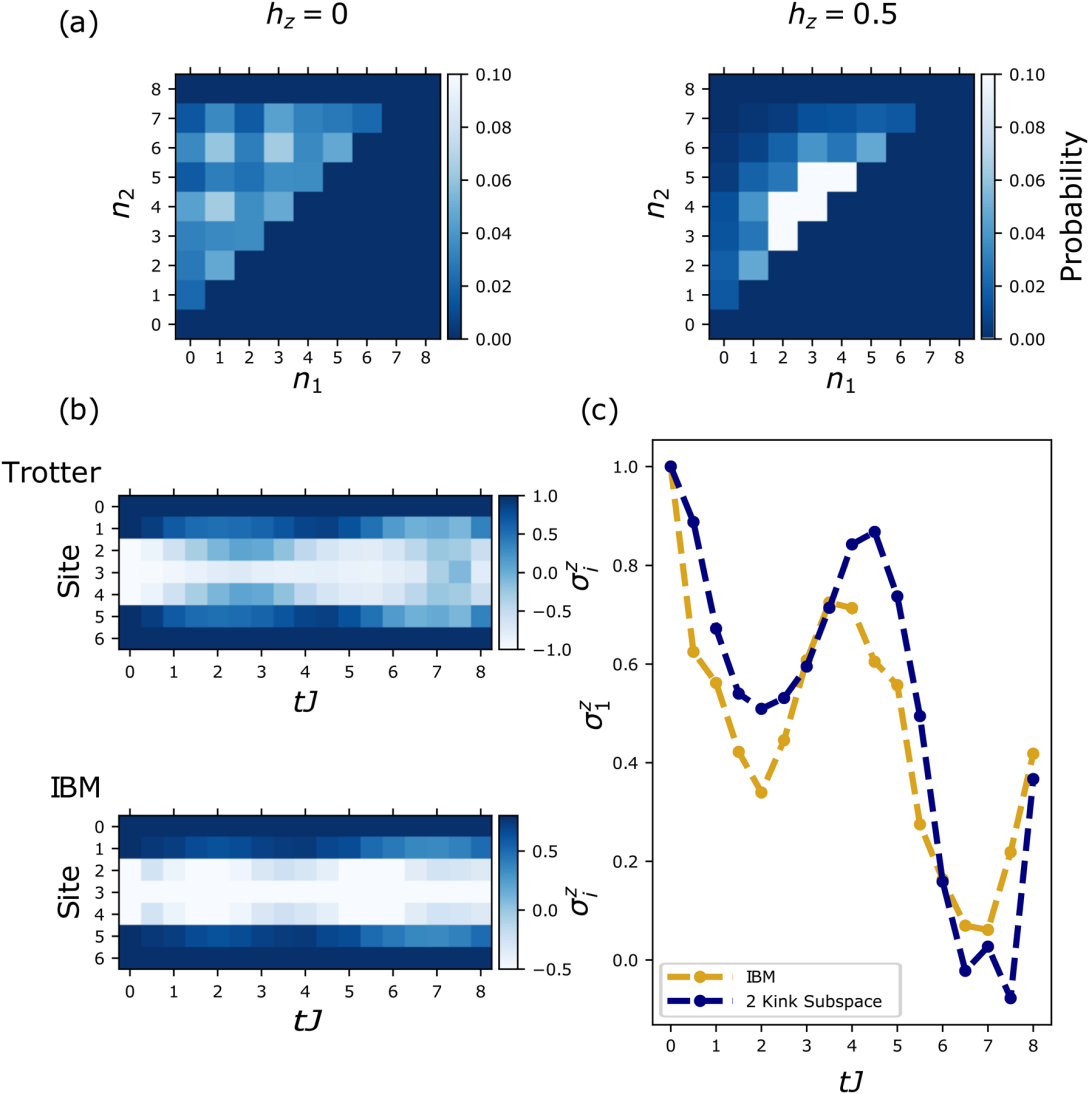
Probability maps and time evolution of local magnetisation. **(a)** Data from the IBM device of the probability maps of kink position after a global quantum quench with and without a longitudinal field (for $h_x=0.5$ and $L=9$) on the state $\vert \frac{L-1}{2},1\rangle$. The graph on the left shows the free kink case, $h_z=0$ and in the graph on the right $h_z =0.5$. Clearly if $h_z=0$, the the kinks have no preference to remain close together as there is no confining field. However, for $h_z = 0.5$, the kinks have a much larger probability to reside close to one another than being separated. **(b)** The local magnetisation after a global quantum quench with $h_x=h_z=0.5$ and $L=7$ on the state $\vert \frac{L-1}{2}-1,3\rangle$. These results show clear oscillatory motion of kinks. This is a high order effect that is only seen with interactions and not just disorder. **(c)** The local magnetisation of the first qubit before symmetrisation, $\sigma _1^z$, is shown explicitly, highlighting the oscillatory behaviour captured by the quantum computer.

(i) In Fig.2 we show results for $\Delta _i^{zz}$ (with $h_z=0, 0.5$ and $h_x=0.5$) from the IBM machine obtained via trotterised time evolution (more details are given in the Supplementary Material) compared to continuous time exact diagonalization (ED) and trotterised ED, both projected to the two kink subspace. The short time dynamics is governed by the free motion of kinks (dashed) before the bound states form and propagate at the slower meson velocity (solid). From these results, initial velocities and subsequent slower velocities were extracted for varying longitudinal fields and compared to theoretically predicted values, which are summarised in Fig.1b. Details how the velocities and error bars were obtained are given in the Supplementary Material. The extracted meson velocities, shown in Fig.1b, match quantitatively the predictions from the two kink subspace analysis.
(ii) The second order Rényi entanglement entropy results are presented in Fig.1c. Here, we compare the exact results calculated via ED and trotterisation with the data from the IBM device. It reproduces the suppression of entanglement spreading that depends on the strength of the confining longitudinal field. We note that the inherent error on IBM devices leads to a constant shift which is removed in Fig.1c, more details are given in the Supplementary Material.
(iii) Finally, Fig.3(a) displays the probability maps of kink motion collected from the quantum computer. These maps show how the longitudinal field favours the two kinks to stay together as expected by confinement dynamics^12^.

To corroborate our findings, it is crucial to confirm that the halting of domain wall spreading for increasing $h_z$ arises from coherent quantum dynamics and not just disorder or noise from the machine which have plagued previous attempts^26^. In Fig.3b we show the evolution of the local magnetisation for a quench with $h_x=h_z=0.5$ and $L=7$. Clear oscillatory patterns of the confined kink motion are observed in Fig.3c which provide direct evidence of higher order interaction effects and not a simple featureless decay of correlations.

## Discussion

We have established that current state-of-the-art quantum computers are able to simulate non-perturbative quantum effects like confinement. Using a specially designed quench set-up has allowed us to show confinement signatures and the formation of domain wall bound states in the paradigmatic TFIM with a longitudinal field. Randomized measurement protocols have enabled us to show the confinement-induced slow-down of entanglement spreading on a digital quantum computer. Next on the agenda will be quantum simulations of confinement effects in spin chains as discussed in relation with scattering experiments of real materials^15,16,29^. On a different front, digital quantum simulations and especially entanglement measurements as presented here will help to further our understanding of non-ergodic quantum dynamics^30^, for example the interplay of confinement and quantum many body scars^19,31^.

Our benchmark results are a crucial step towards the simulation of many-body quantum phenomena beyond the reach of classical computers. The added advantage to similar quantum simulation endeavours in cold atomic gases^17^ or trapped ion quantum simulators^32^ is the ease of initial/final-state preparation/selection, as well as the potential freedom to engineer more complicated theories also in higher dimensions, e.g., for quantum field/gauge theories^7,8^. For example, a next step for digital quantum simulations would be to simulate the Schwinger model in order to observe out-of-equilibrium properties of $1 + 1$ dimensional quantum electrodynamics^6^ before going to higher dimensions. The good news for NISQ limited devices is that signatures of confinement, like pair production or string breaking^33^, are visible already at short times for moderate system sizes. In the long run, a potentially disruptive advantage of digital quantum simulators is the ease of access to experimental hardware. We provide a first example how to use remote access to a NISQ device as a new numerical tool which can perform specific experiments, for example on confinement dynamics, without the need for purpose built set ups^32^.

This work highlights the capabilities of quantum computers in the NISQ era. They can already deliver on Feynman’s original quantum simulation promise — for the time being at least for phenomena like confinement which are observable in intermediate-time dynamics and for moderate system sizes.

While finalising this manuscript, we became aware of a complimentary work^34^ observing confinement dynamics on a trapped ion simulator.

## Supplementary Information

Supplementary Information.

## Data Availability

All data is available upon reasonable request.

## References

[1] Nielsen, M. A. & Chuang, I. *Quantum Computation and Quantum Information* (2002).

[2] Feynman RP (1982). Simulating physics with computers. Int. J. Theor. Phys..

[3] Lloyd, S. Universal quantum simulators. *Science* 1073–1078 (1996). 10.1126/science.273.5278.1073 8688088

[4] Georgescu IM, Ashhab S, Nori F (2014). Quantum simulation. Rev. Mod. Phys..

[5] Kandala A (2017). Hardware-efficient variational quantum eigensolver for small molecules and quantum magnets. Nature.

[6] Martinez EA (2016). Real-time dynamics of lattice gauge theories with a few-qubit quantum computer. Nature.

[7] Jordan SP, Lee KS, Preskill J (2012). Quantum algorithms for quantum field theories. Science.

[8] Zohar E, Cirac JI, Reznik B (2015). Quantum simulations of lattice gauge theories using ultracold atoms in optical lattices. Rep. Prog. Phys..

[9] Preskill J (2018). Quantum computing in the NISQ era and beyond. Quantum.

[10] Brambilla N (2014). QCD and strongly coupled gauge theories: Challenges and perspectives. Eur. Phys. J. C.

[11] Liu, F. *et al.*. *Confined Dynamics in Long-Range Interacting Quantum Spin Chains*. arXiv preprint arXiv:1810.02365 (2018). 10.1103/PhysRevLett.122.150601 PMC6990634 31050545

[12] Fukuhara T (2013). Microscopic observation of magnon bound states and their dynamics. Nature.

[13] McCoy BM, Wu TT (1978). Two-dimensional Ising field theory in a magnetic field: Breakup of the cut in the two-point function. Phys. Rev. D.

[14] Fonseca P, Zamolodchikov A (2003). Ising field theory in a magnetic field: Analytic properties of the free energy. J. Stat. Phys..

[15] Coldea R (2010). Quantum criticality in an Ising chain: Experimental evidence for emergent E8 symmetry. Science.

[16] Lake B (2010). Confinement of fractional quantum number particles in a condensed-matter system. Nat. Phys..

[17] Simon J (2011). Quantum simulation of antiferromagnetic spin chains in an optical lattice. Nature.

[18] Kormos M, Collura M, Takács G, Calabrese P (2017). Real-time confinement following a quantum quench to a non-integrable model. Nat. Phys..

[19] James AJA, Konik RM, Robinson NJ (2019). Nonthermal states arising from confinement in one and two dimensions. Phys. Rev. Lett..

[20] Robinson NJ, James AJ, Konik RM (2019). Signatures of rare states and thermalization in a theory with confinement. Phys. Rev. B.

[21] Rutkevich S (2008). Energy spectrum of bound-spinons in the quantum Ising spin-chain ferromagnet. J. Stat. Phys..

[22] Mussardo G (2011). Integrability, non-integrability and confinement. J. Stat. Mech. Theory Exp..

[23] Cervera-Lierta A (2018). Exact Ising model simulation on a quantum computer. Quantum.

[24] Zhukov A, Remizov S, Pogosov W, Lozovik YE (2018). Algorithmic simulation of far-from-equilibrium dynamics using quantum computer. Quantum Inf. Process..

[25] Francis, A., Freericks, J. & Kemper, A. Quantum computation of magnon spectra. arXiv preprint arXiv:1909.05701 (2019).

[26] Smith, A., Kim, M., Pollmann, F. & Knolle, J. Simulating quantum many-body dynamics on a current digital quantum computer. *npj Quantum Inf.* **5** (2019).

[27] Fagotti M, Calabrese P (2008). Evolution of entanglement entropy following a quantum quench: Analytic results for the X Y chain in a transverse magnetic field. Phys. Rev. A.

[28] Brydges T (2019). Probing Rényi entanglement entropy via randomized measurements. Science.

[29] Wang Z (2018). Experimental observation of Bethe strings. Nature.

[30] Bañuls MC, Cirac JI, Hastings MB (2011). Strong and weak thermalization of infinite nonintegrable quantum systems. Phys. Rev. Lett..

[31] van Voorden, B., Minář, J. & Schoutens, K. Quantum many-body scars in transverse field Ising ladders and beyond. arXiv preprint arXiv:2003.13597 (2020).

[32] Becker, P. *et al.* Observation of domain wall confinement and dynamics in a quantum simulator. *Bull. Am. Phys. Soc.* (2020).

[33] Magnifico, G. *et al.*, Real time dynamics and confinement in the Zn schwinger-weyl lattice model for 1+ 1 qed. arXiv preprint arXiv:1909.04821 (2019).

[34] Tan, W. *et al.*, Domain-wall confinement and dynamics in a quantum simulator. *Nat. Phys.* 1–6 (2021).

[35] Syljuåsen OF (2015). Dynamical structure factor of magnetic Bloch oscillations at finite temperatures. Eur. Phys. J. B.

[36] Lagnese G, Surace FM, Kormos M, Calabrese P (2020). Confinement in the spectrum of a Heisenberg-Ising spin ladder. J. Stat. Mech. Theory Exp..

[37] Vovrosh, J. *et al.**Simple Mitigation of Global Depolarizing Errors in Quantum Simulations*. arXiv preprint arXiv:2101.01690 (2021). 10.1103/PhysRevE.104.035309 34654120

