# Supplementary material for: Confinement and entanglement dynamics on a digital quantum computer
Source: Sci Rep. 2021 Jun 2;11:11577. doi: 10.1038/s41598-021-90849-5 (PMC8172933; doi:10.1038/s41598-021-90849-5)
Supplement: Supplementary file 1 — Supplementary Information. [file 41598_2021_90849_MOESM1_ESM.pdf]

Methods and Supplementary Material

Calculation of the meson velocities.— In order to calculate the meson velocities we proceed by taking the Fourier transform of Eq.(2) over j, $|k,n\rangle = \frac{1}{\sqrt{L}}\sum_j \exp\bigl(-ik\frac{n}{2} - ikj\bigr)\,|j,n\rangle$, obtaining

$$\mathcal{H} = \sum_{k,n}\bigl[V(n)\,|k,n\rangle\langle k,n| + 2h_x\cos\frac{k}{2}\bigl(|k,n\rangle\langle k,n-1| + |k,n\rangle\langle k,n+1|\bigr)\bigr] \quad (1)$$

which, for an infinite chain, can be diagonalised using the transformation

$$|k,\alpha\rangle = \sum_n C_\alpha \mathcal{J}_{n-\nu_{k,\alpha}}(x_k)\,|k,n\rangle\,. \quad (2)$$

in which $\nu_{k,\alpha} = \frac{E_{k,\alpha}}{2h_x}$, $x_k = \frac{2h_z\cos\frac{k}{2}}{h_x}$, $\mathcal{J}$ is the Bessel function of the first kind and the coefficient C_α is used for normalisation (*1*). Bessels functions can also diagonalise the finite system, (*2*), but as the boundary effects for our protocols are very small, the analytics for an infinite chain are sufficient. The energy levels $E_{k,\alpha}$ can be computed via the boundary condition that $\mathcal{J}_{-\nu_{k,\alpha}}(x_k) = 0$. Continuing with the analogy from QCD, the masses of the mesons formed by the domain wall pairs can be calculated via the difference between energy levels and the ground state. As well as these, the allowed velocities of the mesons can be calculated as the maximal gradient of each energy level, see Fig.1(b) in the main text. Note that, as we only consider the motion of very small mesons ($n = 1$), we are not troubled by finite size affects when measuring their velocities and can directly compare them to the theoretical predictions which we have also checked via ED (*2*).

The observation of these quantities then provides direct evidence of the underlying confinement dynamics. For example, in Fig 1 (a) in the main text, with $h_z = 0$ one velocity is seen and corresponds to the free kink motion described by the TFIM. However, if $h_z = 0.2$ there are two velocities. The initial velocity from $t = 0$ until $t \sim 4J$ again shows free kink motion, while for times $t > 15J$ the slower velocity of the meson governs the dynamics.

Implementation on the IBM quantum computer.— In order to implement quench dynamics onto a quantum computer one must decompose the time evolution operator, $U(t) = e^{-iHt}$ into one and two qubit gates. To accomplish this, the standard Suzki-Trotter decomposition, commonly known as trotterisation, is used. Trotterisation discretises time based on the fact that for two non-commuting operators A and B, $e^{A+B} = \lim_{n\to\infty}\left(e^{\frac{A}{n}}e^{\frac{B}{n}}\right)^n$. Using this for a Hamiltonian of the form $H = A + B$, the time evolution operator can be written as

$$U(t) = e^{-iHt} = e^{-iAt}e^{-iBt} + O(t^2). \quad (3)$$

Clearly, the Suzki-Trotter decomposition will only give reliable results for small times. Thus, in order to simulate long time periods on must use multiple trotter steps, $U(t) \sim U(\Delta t)$ $U(\Delta t)$ $U(\Delta t)$..., here $U(\Delta t)$ is given by the approximation in Eq.(8) The number of trotter steps required depends on the length of time that is being simulated. There are extensions to this approximation to further reduce the resulting error. Building on our previous work, we use the symmetric decomposition (*3*) given by:

$$U(t) = e^{-iHt} = e^{-ia\frac{t}{2}}e^{-ibt}e^{-ict}e^{-ia\frac{t}{2}} + O(t^3), \quad (4)$$

with $a = -Jh_x\sum_{i=1}^{L}\sigma_i^x$, $b = -Jh_z\sum_{i=1}^{L}\sigma_i^z$ and $c = -J\sum_i^L\sigma_i^z\sigma_{i+1}^z$. Note that e^{-ibt} is not symmetrised in this expression as $[b, c] = 0$. A schematic of the gate sequence required to implement this is given in Fig.1.

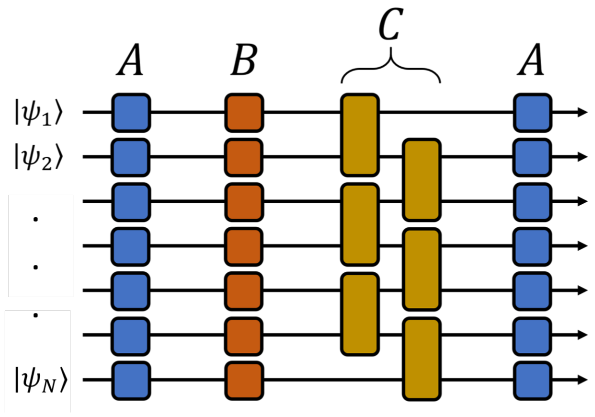

Figure 1: **The gate sequence in each trotter step performed on the quantum computer**. Here, $A = e^{\frac{itJh_x}{2}\sum_{i=1}^{L}\sigma_i^x}$, $B = e^{itJh_z\sum_{i=1}^{L}\sigma_i^z}$ and $C = e^{itJ\sum_i^L \sigma_i^z\sigma_{i+1}^z}$.

Velocity extraction.— In order to extract the initial free kink and later meson velocities from the mitigated data obtained by the IBM device the gradient of the light cone formed in Δ_i^{zz} data is computed. Due to the inherent error in the NISQ device, there is a level of uncertainty in the saturation levels that should be used. Thus, a range of velocities are computed and the averages and standard deviations are presented in Fig.1 in the main text.

To attain the initial velocities, data for quench dynamics up to $t = 4J$, a time before boundary effects present on the kinks, was simulated using four trotter steps. In these results there are initialisation errors that would cover up the free kink velocity unless they are removed. Fig.2 shows Δ_i^{zz} data collected for sites $i \in \{0, 1, 2\}$ after forcing the minimum of each to be zero, this removes the effect of the initialisation error. To calculate a velocity one can choose a saturation level, S, and find the times at which Δ_i^{zz} surpasses this number for each site $i \in \{0, 1, 2\}$. This gives three (x, t) coordinates for which the gradient of a line of best fit gives the velocity. When computing the light cone gradients for initial velocities the same range of saturation levels were used for each value of h_z for consistency.

It turns out that the meson velocities are much easier to obtain because the meson velocities themselves are observed at a larger scale. Data was collected up to $t = 8J$, a time such that boundary effects are not present, using seven trotter steps. An initial time of $t = 4J$, roughly the time at which mesons form, was used as the starting point of the light cone to measure the meson velocities. The light cones produced for a given saturation level, $S = 0.21$, and the corresponding velocities are shown in Fig.3. Again, the same range of saturation levels was used for each value of h_z to obtain an average velocity.

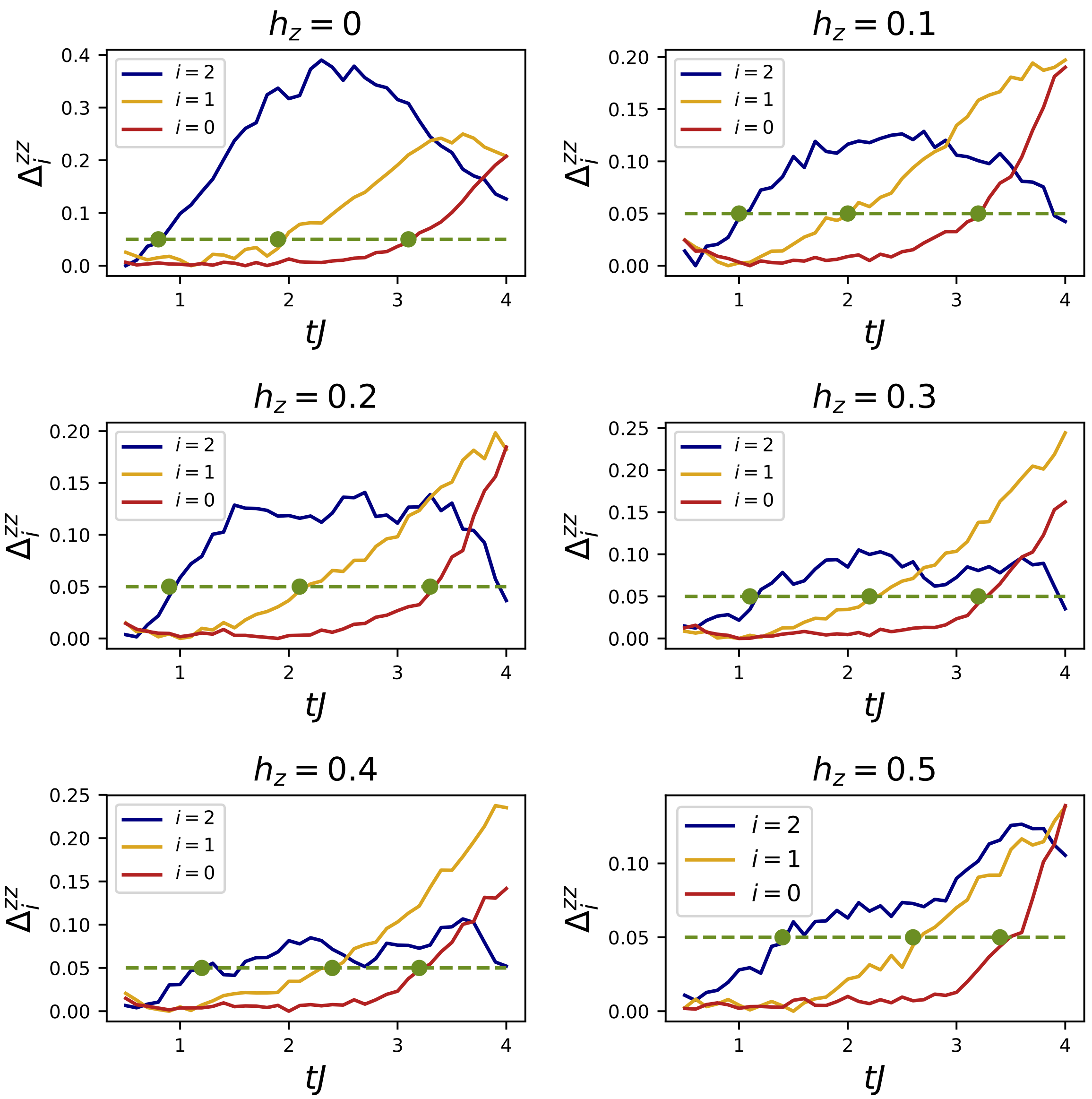

Figure 2: Δ_i^{zz} **data used to calculated initial velocities of kinks**. Re-scaled data for Δ_i^{zz} after a global quantum quench to the TFIM with a longitudinal field on the state $\left|\frac{L-1}{2}, 1\right\rangle$. In all presented data $h_x = 0.5$ and $L = 9$. Green points correspond to the (x, t) coordinates used to calculate the initial velocities for a saturation level of $S = 0.05$. Each figure shows clearly the free kink movement from sites 2 to 0. The velocities measured depend heavily on the saturation levels used hence the use of a large range to get an average.

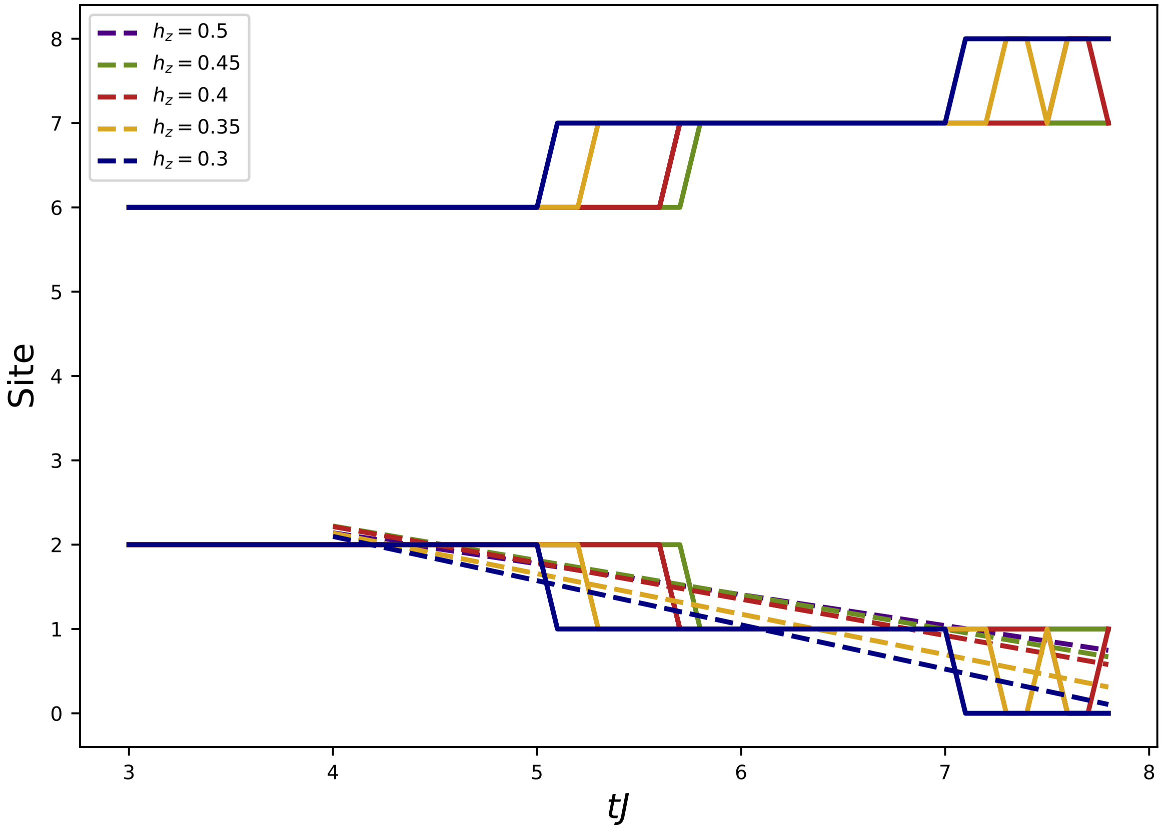

Figure 3: **Light cones produced by saturating Δ_i^{zz} data**. Data saturated at $S = 0.21$ for Δ_i^{zz} after a global quantum quench to the TFIM with a longitudinal field on the state $\left|\frac{L-1}{2}, 1\right\rangle$. In all presented data $h_x = 0.5$, and $L = 9$. Clear light cones are formed with varying velocities that depend on the strength of the longitudinal field.

Validity of the two kink projection.— In all of the results presented for Δ_i^{zz}, probability maps and local magnetisation, we employed the error mitigation of post selecting the data that lie within the two kink subspace. In this section, we discuss the validity of the method. To quantify to what extent the two kink subspace dictates the motion of kinks one can look at $\phi(t)$ defined as

$$\phi(t) = \sum_i |\langle i|\psi(t)\rangle|^2. \tag{5}$$

Here, $|\psi(t)\rangle$ is the the full many-body wave function of the spin chain at the time t after the global quantum quench and $\{ |i\rangle \}$ are the basis states of the two kink subspace. From this definition of ψ, $\phi(t)$ can be seen as a measure of the percentage of the full wave function, $|\psi(t)\rangle$, that remains in the two kink subspace. In Fig.4 it is clear that the two kink subspace is responsible for $> 80\%$ of the dynamics in the protocols used in this paper. Hence, by post selecting states measured in the two kink subspace the correct states will be selected up to reasonable error. Beyond this, in Fig.5 a comparison of the results taken from the IBM device before and after error mitigation are presented. This highlights the effect of the subspace projection as well as enforcing the inversion symmetry of the initial state in order to project away background noise and extract the desired physical results. The power of this projection can be understood via the following argument. Let δ be the probability of measurement error for a qubit. For a two kink state there are just four possible erroneous spin flips that do not result in the measurement to be outside the subspace. Therefore, to first order approximation the error that will not be mitigated is just 4δ which does not scale with system size. In turn, the probability of error in simulations that can be mitigated via a projection into the two kink subspace is $(N-4)\delta$. At second order, the probability of two consecutive errors occurring that take the result out of and then back into the subspace is of order δ^2. With a small δ this second order process is much less likely, and thus, this projection allows large error mitigation.

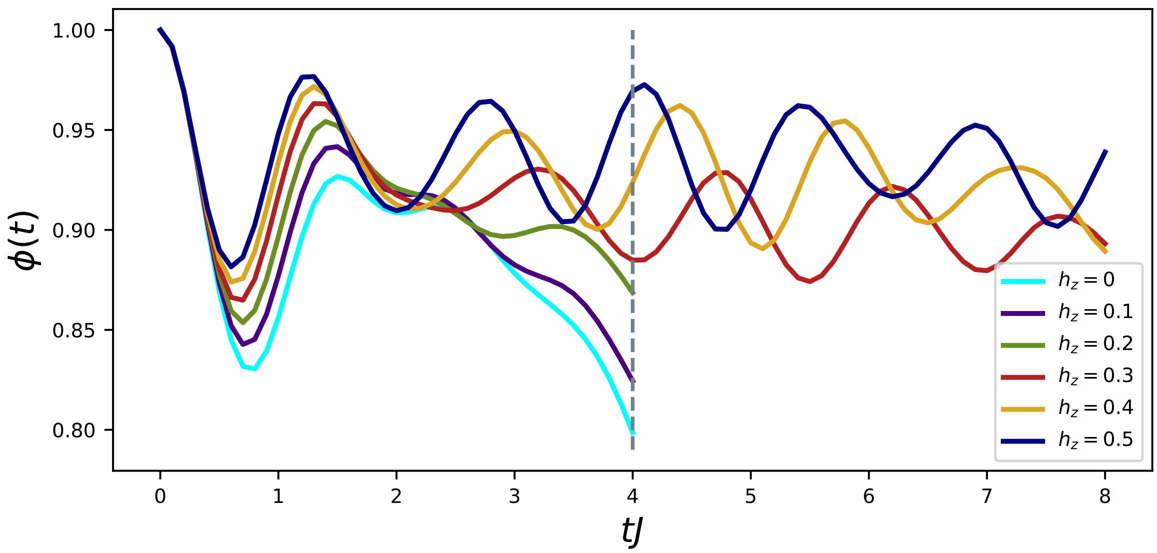

Figure 4: **A measure of the contribution of the two kink subspace**. $\phi(t)$ after a global quantum quench to the TFIM with a longitudinal field on the state $\left|\frac{L-1}{2}, 1\right\rangle$. Here $h_x = 0.5$ and $L = 9$. As only initial velocities were calculated for $h_z < 0.3$, $\phi(t)$ is presented in the range $tJ < 4$ for these longitudinal field strengths, this is highlighted by the horizontal dashed line. Even in the free kink case, $h_z = 0$, the majority of the dynamics, $> 80\%$, is within the two kink subspace for the times used to compute the velocities in Fig.1 (b) in the main text.

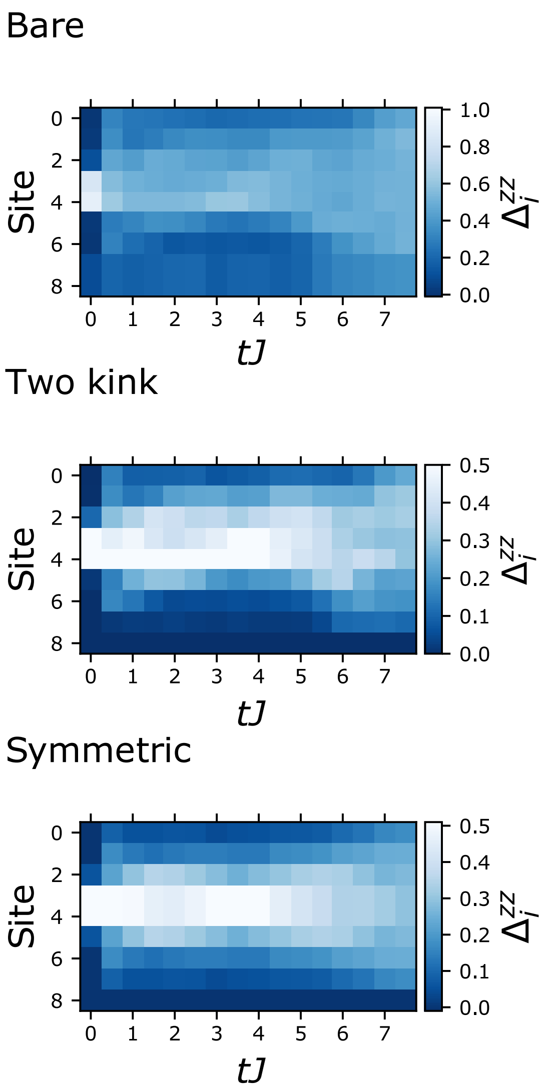

Figure 5: **A comparison of result collected from the IBM device with and without error mitigation**. The IBM results for Δ_i^{zz} presented in Fig.2 in the main text with $h_x = 0.5$, $h_z = 0.5$ and $L = 9$. The bare result are shown first followed by the same data after post selection of the two kink subspace states, and finally the symmerised results. The background noise is considerably reduced by the two kink subspace projection and the light cones are restored by the process of forcing the inversion symmetry of the initial state. This allows the confinement physics to be observed and the gradients of light cones to be measured.

Rényi entropy measurements.— Although the qualitative structure of Rényi entanglement entropy obtained on the IBM machine is correct, in order to obtain quantitative agreement with ED results a constant shift is needed. As the circuit depths used are too low for coherence times to be the source of this problem, the two possible causes are initialisation and measurement errors or gate errors. To understand this we performed two separate tests.

Firstly, we ran a simple circuit in which we directly measure the entropy of the system initialised in the $|\uparrow\uparrow\downarrow\downarrow\uparrow\uparrow\rangle$ state with the expected result of zero. On the IBM machine this is equivalent to starting a run, initialising the state and adding the random single qubit gates on the half chain in consideration and measuring. As errors in single qubit gates are very low, any error in the entropy calculation can be assigned to initialisation and measurement error with a high degree of certainty. Using the built in measurement mitigation tool in Qiskit Ignis the entropy recorded is ~ 0 as seen in Fig.6. Thus, we conclude that this shift is not an effect of initialisation and measurement error.

Secondly, we ran circuits with a varying number of trotter steps that only evolve the state to very short times, $tJ = 0.01$. This will also have an expected Rényi entropy of zero to the degree of accuracy which is obtainable by the IBM device. This protocol will include gate errors as well as initialisation and measurement errors. From Fig.6 it can be seen that the error in performing gates on the IBM device has the effect of a constant entropy shift observed for all times measured on the device that grows with the number of trotter steps used. As this shift is not time dependent it is reasonable to assume it is due to errors in implementation only and can be safely removed to observe the true physics. In fact, a basic error model can be derived to account for this shift in random measurement protocols which will be presented elsewhere (*4*).

As well as removing this shift, in order to obtain clear results for entropy, a system size of six sites was used. It is thus important to rule out finite size effect as the cause of the suppression in entropy. Fig.7 shows the finite size scaling of Rényi entropy. For a system size of six times

up to $tJ \sim 2.5$ are free of finite size effect. Although for longer times slight effects of a short chain are visible when considering longitudinal field strengths of $h_z = 0$ and 0.5, they are small enough for the halting of entropy growth due to confinement to be visible. Furthermore, there are no finite size effects for a longitudinal field of strength $h_z = 0.75$. Hence, the suppression seen in the IBM device can be confidently assigned to confinement dynamics.

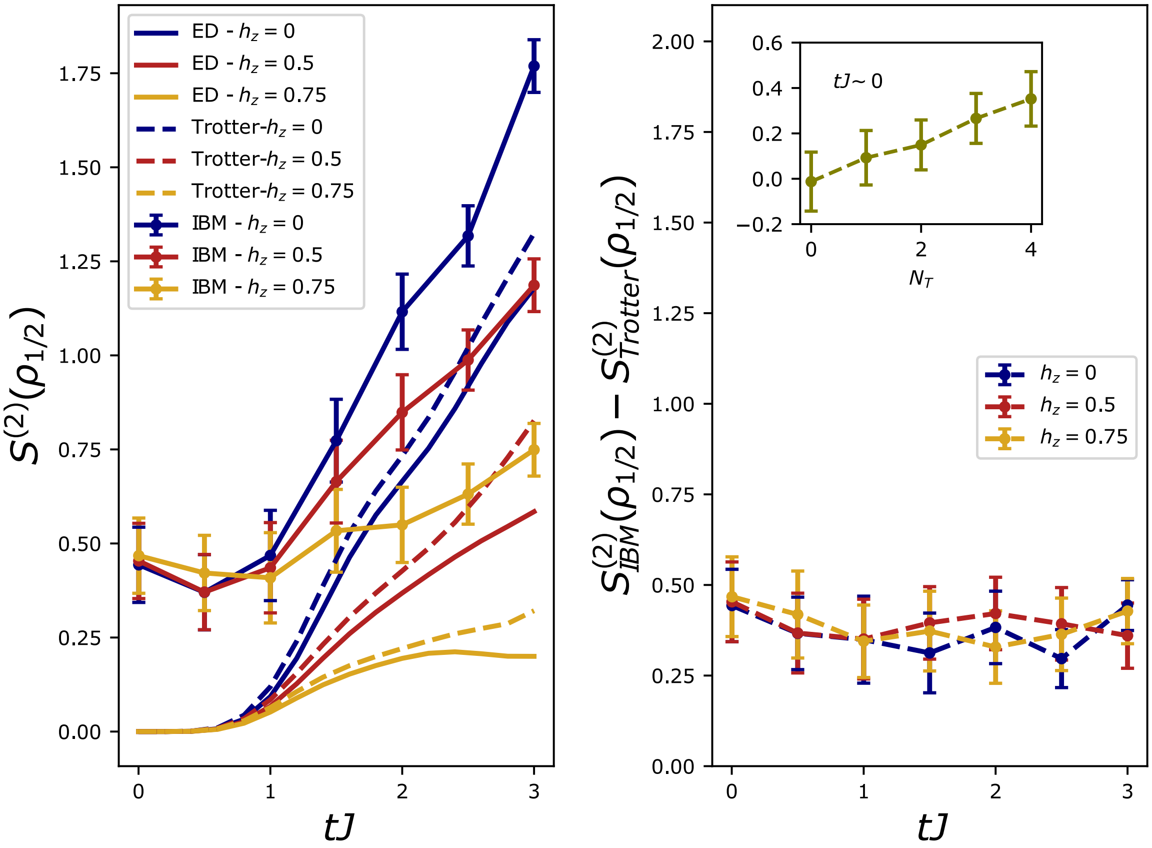

Figure 6: **Time evolution of the half chain Rényi entropy without shift mitigation**. The left panel shows the data for the second order Rényi entropy presented in Fig.1(c) in the main text with the constant shift from the IBM device left unresolved. The right panel shows explicitly this constant shift due to gate error with the inset plot showing how this shift depends on the number of trotter steps, N_T. This shift is time independent and grows with N_T, hence it is a results of implementation error on the IBM device.

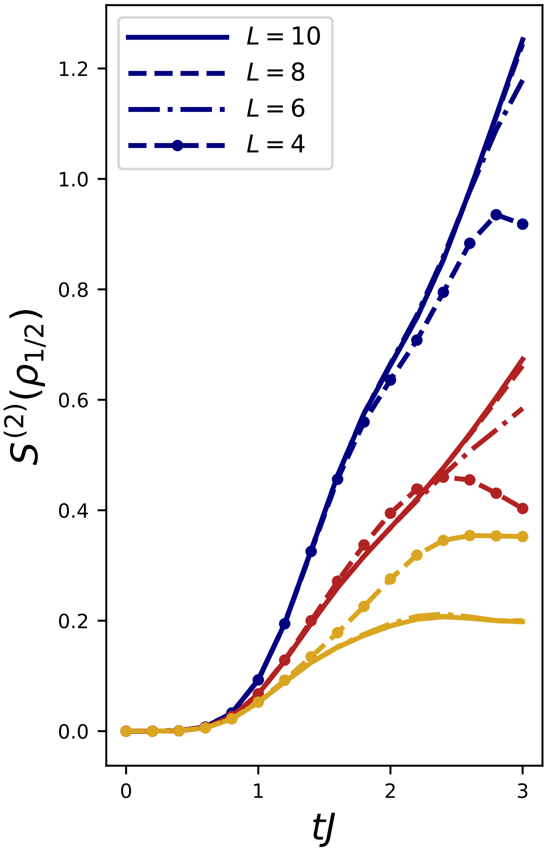

Figure 7: **Finite size scaling for the time evolution of the half chain Rényi entropy**. ED data for the second order Rényi entropy after a global quantum quench to the TFIM with varying longitudinal field strengths on the state $\left|\frac{L}{2} - 1, 2\right\rangle$ such that $L \in \{4, 6, 8, 10\}$. Here, $h_x = 0.5$. Blue results correspond to $h_z = 0$, red results correspond to $h_z = 0.5$ and gold results correspond to $h_z = 0.75$. Although finite size effects are present for a chain of length $L = 6$ they only have a slight effect for times $tJ > 2.5$ and are not large enough to hide the halting effect of entropy growth due to confinement.

References

1. O. F. Syljuåsen, *The European Physical Journal B* **88**, 252 (2015).
2. G. Lagnese, F. M. Surace, M. Kormos, P. Calabrese, *Journal of Statistical Mechanics: Theory and Experiment* **2020**, 093106 (2020).
3. A. Smith, M. Kim, F. Pollmann, J. Knolle, *npj Quantum Information* **5** (2019).
4. J. Vovrosh, *et al.*, *arXiv preprint arXiv:2101.01690* (2021).
